# Bioimpedance in neurosurgery for tumor margin delineation

**DOI:** 10.1016/j.bas.2025.105894

**Published:** 2025-12-03

**Authors:** Jakub Petrzelka, Martin Rozanek, Martin Cerny, David Netuka

**Affiliations:** a Department of Neurosurgery and Neurooncology, First Faculty of Medicine, Charles University and Military University Hospital, Prague, Czech Republic; b Department of Biomedical Technology, Faculty of Biomedical Engineering, Czech Technical University in Prague, Kladno, Czech Republic

**Keywords:** Bioimpedance, Brain tumor margins, Glioma, Intraoperative monitoring, Neurosurgery

## Abstract

**Introduction:**

The electrical properties of brain tissue, shaped by cellular composition, fluid content, and ion distribution, create measurable impedance differences that can be exploited intraoperatively [1–7]. Bioimpedance has emerged as a promising adjunct for guiding resection in gliomas and other brain lesions, offering real-time information beyond microscopic views, fluorescence, or MRI imaging.

**Research question:**

This review synthesizes current evidence to assess whether bioimpedance can reliably delineate tumor margins in neurosurgery, evaluating differences between normal and pathological tissue and its potential for routine use.

**Material and methods:**

We reviewed key studies (2014–2024) on bioimpedance in brain tissue, focusing on in vivo measurements, systematic analyses, and oncology applications, selected from PubMed and Scopus for relevance to intraoperative margin identification. Keywords included bioimpedance, brain tumor margins, glioma surgery, intraoperative monitoring, and neurosurgery. Two recent reviews by Georgiannakis et al. (2024) and Abboud et al. (2022) were included to provide up to date insights.

**Results:**

Recent studies confirm tumor tissue shows distinct resistivity compared with surrounding white and gray matter [1–3, 5, 8]. For example, Abboud et al. (2022) reported white matter at 13.3 ± 1.7 Ω m, peritumoral edema at 8.5 ± 1.6 Ω m, low-grade gliomas at 6.4 ± 1.3 Ω m, and high-grade gliomas at 5.0 ± 1.0 Ω m (enhancing) or 3.9 ± 1.1 Ω m (necrotic; p < 0.001). Though absolute values vary, differences between physiological white/gray matter and tumor remain consistently significant.

**Discussion and conclusion:**

Methodological heterogeneity and lack of standardization prevent routine clinical application. Standardized protocols and larger-scale validation are needed to facilitate bioimpedance's role in decisions about the radicality of resection.

## Highlights

• Bioimpedance can detects differences between tumor and brain tissue, or in general between healthy tissue and tumour.
• Gliomas, edema, and necrosis show difference in resistivity, while measuring intraoperatively.
• Bioimpedance could be additional technique used for decisions about radicality of resection
• Lack of standardization and wider use limits current clinical adoption.

## 1 Introduction

Diffuse gliomas infiltrative grow behavior represents a challenge for maximal safe resection. Their infiltrative growth extends beyond radiographic or visual boundaries, leaving residual microscopic tumor, despite aggressive surgery (Louis et al., 2021; Smith et al., 2022; van den Bent et al., 2023). Gross total resection, or near total resection strategies are used for improving patient outcome, yet avoiding neurological deterioration caused by surgery. This may be difficult at tumor margins, where borders between tumor and brain tissue may not be sufficiently visible, despite use of adjuncts such as 5-ALA fluorescence, intraoperative MRI or ultrasound to improve visualization. Those remain helpful, yet imperfect at detecting microscopic infiltration.

Bioimpedance provides an additional way to characterize tissue, measuring its opposition to alternating current through ionic conduction and cell membrane capacitance. For this purpose, bipolar or monopolar probes with reference electrode are used. These properties are reported as either resistivity (Ω·m) or conductivity (S/m), obtained from measurements between the two electrode poles. Difference in tissue composition, for instance tumor or edema, consistently alter these properties by changes in cellularity, disrupting membranes and increasing extracellular water, creating measurable contrast with normal brain tissue (Geddes et al., 1967; Meroni et al., 2016; Ward et al., 2013).

Bioimpedance is already established in body composition analysis and has been explored in breast, prostate, cervical, and skin cancers, where malignant tissue differs from benign. In neurosurgery, interest has accelerated over the past decade, with in vivo studies confirming reproducible impedance differences between normal parenchyma, edema, and tumor. This review synthesizes current evidence of intraoperative measurements.

## 2 Material and methods

### 2.1 Principles of electrical impedance

Bioimpedance measures the resistance of brain tissue to alternating current, driven by ion-rich fluids and the capacitive effect of cell membranes, varying with frequency. The impedance Ζ is given by:

$$Ζ=R+\frac{1}{j\omega C}$$

where R is resistance (Ω), C is capacitance (F), ω is angular frequency (rad/s), and j is the imaginary unit. It is reported as resistivity (*ρ*, Ω·m) or conductivity (*σ*, S/m), where *σ= 1/ρ,* with conversion depending on electrode geometry (*ρ=Z. A/L,* where *A* is area and *L* is length).

The frequency of the applied AC significantly influences impedance measurements, reflecting distinct tissue properties across the spectrum. At low frequencies (<1 kHz), current is predominantly restricted to extracellular fluid pathways due to the high capacitive impedance of cell membranes. This range is particularly sensitive to edema and extracellular fluid volume, offering insights into peritumoral swelling. The intermediate frequency range, known as the β-dispersion (10–100 kHz), is where membrane capacitance is partially overcome, allowing current to penetrate intracellular spaces. This range, peaking around 50 kHz, is most relevant for tumor margin studies, as it captures cell density, membrane integrity, and fluid shifts. The Cole-Cole model, $Ζ\left(f\right)=R_{\infty }+\frac{R_{0}-R_{\infty }}{{1+\left(j\omega \tau \right)}^{\alpha }}$, approximates this frequency-dependent behavior, where $R_{0}$ and $R_{\infty }$ are low- and high-frequency resistances, τ is the relaxation time constant, and α (0 < α ≤ 1) adjusts for relaxation time distribution (Geddes et al., 1967; Meroni et al., 2016; Ward et al., 2013; Gregory et al., 2012).

## 3 Bioimpedance measurements in brain tissue

Several studies provide robust evidence for bioimpedance's role in tumor margin identification. Koessler et al. (2017) conducted 1421 in vivo intracerebral measurements in 15 epilepsy patients using multicontact depth electrodes with a 50 kHz current injection. Conductivity was estimated with numerical modeling around each electrode contact pair. Gray matter measured 0.26 S/m (≈385 Ω cm), white matter 0.17 S/m (≈590 Ω cm), and epileptogenic zones 0.29 S/m (≈345 Ω cm). Differences between gray and white matter were highly significant (p < 0.0001), and epileptogenic zones showed significantly higher conductivity than healthy gray matter (p = 0.012), even when MRI was normal (p = 0.005). These data established robust reference values for physiological tissue and confirmed measurable electrical contrasts in pathological zones (Koessler et al., 2017).

Latikka et al. (2019) performed calibrated in vivo resistivity measurements in 20 patients using a monopolar probe delivering a 50 kHz sinusoidal current. Tumor types included meningiomas (n = 8), low-grade gliomas (n = 4), and high-grade gliomas (n = 5), along with a other lesions. Mean resistivity values were 530 Ω cm for meningiomas, 160 Ω cm for low-grade gliomas, and 498 Ω cm for high-grade gliomas (Louis et al., 2021). Differences between high- and low-grade gliomas, as well as between meningiomas and low-grade gliomas, were statistically highly significant (p < 0.001 and p < 0.004, respectively), whereas meningiomas and high-grade gliomas did not differ significantly. Gray matter measured on average 306 Ω cm, and white matter 372 Ω cm (Latikka et al., 2019).

Abboud et al. (2021) study, including 92 patients (diffuse gliomas CNS WHO 2 n = 16, CNS WHO 3 n = 10, CNS WHO 4 n = 33 and metastases n = 33) and using a calibrated bipolar stimulation probe at 140 Hz, showed resistivity of white matter outside peritumoral edema measured 13.3 ± 1.7 Ω m, compared with 8.5 ± 1.6 Ω m within edema. Gliomas WHO grade 2 and 3 showed mean resistivity values of 6.3 and 6.4 Ω m, ±1.3 and 0.9 Ω m, while glioblastomas (WHO 4) measured 5.0 ± 1.0 Ω m in enhancing regions and 3.9 ± 1.1 Ω m in necrosis. Metastases averaged 5.4 ± 1.3 Ω m. Importantly, no overlap was observed between white matter and tumor resistivity values within the same patient. On average, white matter values were ∼158 % higher than tumor, and edema values ∼85 % higher than tumor, underscoring the robustness of the contrast despite intra- and interpatient variability (Abboud et al., 2021a).

Two reviews have recently addressed the role of impedance in brain tumor surgery. Abboud et al. (2021) provided a mini-review, summarizing methodological principles and feasibility data from early impedance studies. They highlighted consistent tumor-normal brain tissue differences but emphasized the lack of standardization and clinical integration (Abboud et al., 2021b).

Georgiannakis et al. (2024) expanded on this work with a systematic review of 18 studies including 286 patients, evaluating both impedance and electrocorticography (ECoG) for intraoperative margin identification.

Electrocorticography (ECoG) studies (n = 8) showed consistent electrophysiological alterations at tumor margins, particularly increased high-gamma power and connectivity changes. No study has yet integrated impedance and ECoG in a single device, though Georgiannakis et al. identified this as a promising future direction (Georgiannakis et al., 2024).

## 4 Broader use in oncology

Across other oncological surgeries, bioimpedance also shown value in differentiating tumor tissue from surrounding healthy structures. Pathiraja et al. (2020) systematically reviewed 7035 patients across 51 studies and 16 different cancer types. Their synthesis confirmed consistent malignant-benign impedance differences (Pathiraja et al., 2020).

Breast cancer: Twenty-one studies (n = 4056) patients reported high diagnostic accuracy of impedance spectroscopy for differentiating malignant from benign lesions. Several intraoperative studies explored margin assessment, with AUROC values up to 0.93. Handheld probes were shown to identify residual tumor not detected by frozen section.

Prostate cancer: Nine studies (n = 573) demonstrated impedance differences between malignant and benign tissue, some correlating with Gleason score. Both ex vivo needle-based measurement and intraoperative applications were tested.

Cervical cancer: Eleven studies (n = 1667) consistently reported significant impedance differences in cervical intraepithelial neoplasia (CIN) and carcinoma. Multicenter studies validated impedance as a screening adjunct, showing sensitivity and specificity comparable to cytology.

Skin cancer: Seven studies (n = 428) evaluated electrical impedance spectroscopy in melanoma and non-melanoma skin cancers. Devices achieved reproducible discrimination between malignant and benign lesions and are already in commercial use for dermatological assessment.

Oral cavity cancers: Four studies (n = 164) demonstrated consistent differences between dysplastic, malignant, and normal mucosa. Impedance showed potential as a non-invasive diagnostic and intraoperative margin tool.

In gastrointestinal, liver, bladder, and ovarian cancers, only small feasibility series (typically <50 patients) have been published. These indicate diagnostic potential, but differences in technique make the findings difficult to compare.

## 5 Discussion

The electrical properties of brain tissue, shaped by cellular structure, fluid distribution, and ion mobility, allow bioimpedance to highlight clear differences between tumor and healthy tissue, making it a valuable intraoperative aid. Recent research demonstrates significant impedance variations across gray matter, white matter, edema, and different tumor types, but the number of publications is still limited.

This suggests bioimpedance could enhance resection accuracy, particularly for gliomas, by providing real-time insights alongside 5-ALA fluorescence or iMRI, which often miss microscopic tumor spread, and offering a solution in cases where these tools are unavailable or less effective, such as with 5-ALA in low-grade gliomas (LGG) (van den Bent et al., 2023; Millesi et al., 2020; Sedlak et al., 2025). The consistent differences, despite varying absolute values, support its potential as a margin marker, though no standard threshold has been established due to differing methods.

Despite its potential, bioimpedance is not yet widely used in neurosurgery or other oncology fields. Inconsistent results, driven by variations in technique, such as electrode setup, frequency choice, and factors like temperature or irrigation during measurement, can skew readings and undermine reliability.

## 6 The BRAIN-IMP study

Building on current evidence, we aim to investigate brain bioimpedance further and evaluate its potential role in routine neurosurgical practice.

Published investigations of brain-tumor impedance have used a variety of measurement setups, including monopolar and bipolar electrode systems, as well as differing stimulation parameters such as current amplitude and excitation frequency. Most of these reports have been exploratory feasibility studies, aiming primarily to demonstrate impedance differences between various tissue types. We hypothesize that the sensitivity and discriminatory power of impedance measurements are strongly frequency-dependent, and that the optimal frequency range may also vary with tissue type due to differences in cellular integrity, particularly alterations in cell-membrane structure. To test this hypothesis, we will apply a broader spectrum of excitation frequencies, spanning 10–100 kHz, to systematically evaluate their influence on tissue differentiation as a function of cell-wall damage and related pathological changes.

In addition, we will investigate how electrode selection and spatial orientation affect the accuracy, reproducibility, and spatial specificity of the impedance measurements. This analysis will help us to identify electrode configurations that minimize artefacts and maximize sensitivity for intraoperative or diagnostic applications.

For this purpose, we will rely primarily on equipment already routinely established for intraoperative electrophysiological monitoring, together with bipolar probes. These devices include an impedance measurement function, which is typically used to confirm correct electrode placement. The benefit we see in evaluating this setup is no additional cost for devices already in use. The key questions and points of focus are:

1. **Reference values:** Resistivity of healthy gray and white matter
2. **Tumor:** Diagnosis, grade, molecular profile, edema, prior treatment, MRI navigation correlation, location
3. **Surgical context:** Irrigation fluid, CSF/blood pooling
4. **Measurement parameters and instrumentation:** Frequency range, electrode type and geometry, spacing, calibration
5. **Environment:** Local temperature, contamination, irrigation conditions.
6. **Tissue labeling:** Histopathology confirmation, co-registration with adjuncts (5-ALA, iMRI, preop.MRI navigation).
7. **Outputs:** Raw impedance and converted resistivity/conductivity
8. **Histopathology:** Categorize tumors based on their cellularity
9. **Anesthesia/physiology:** Brain temperature, blood pressure, mannitol, electrolytes

## 7 Conclusion

The electrical properties of brain tissue, shaped by cellular composition, fluid content, and ion distribution, create measurable impedance differences that can be exploited intraoperatively. Measurement of brain tissue resistivity has emerged as a promising adjunct for guiding resection in gliomas and other brain lesions, offering additional real-time information beyond microscopic views, fluorescence, or MRI imaging. Recent clinical studies, including systematic reviews, confirm that tumor tissue demonstrates different resistivity compared with surrounding white and gray matter. Though absolute impedance values for specific tissues vary considerably between studies, the difference between physiological white matter, gray matter, and tumor has been consistently significant, while no consensus exists on thresholds for intraoperative use. Despite supportive evidence for bioimpedance use as a tumor margins marker, methodological heterogeneity and lack of outcome-linked trials prevent routine clinical application. Standardized protocols and validation on a larger scale should be established to help evaluate benefits of impedance measurement use during surgery and its adoption it in practice.

## Declaration of competing interest

The authors declare that they have no competing interests.
